# Phage-Based Control of Methicillin Resistant *Staphylococcus aureus* in a *Galleria mellonella* Model of Implant-Associated Infection

**DOI:** 10.3390/ijms232314514

**Published:** 2022-11-22

**Authors:** Alessandro Materazzi, Daria Bottai, Claudia Campobasso, Ann-Brit Klatt, Novella Cesta, Margherita De Masi, Andrej Trampuz, Arianna Tavanti, Mariagrazia Di Luca

**Affiliations:** 1 Department of Biology, University of Pisa, 56127 Pisa, Italy; 2 Department of Biosystems, KU Leuven, 3001 Leuven, Belgium; 3 Department of Internal Medicine/Infectious Diseases and Pulmonary Medicine, Charité-Universitätsmedizin Berlin, 13353 Berlin, Germany; 4 PhD Course in Microbiology, Immunology, Infectious Diseases and Transplants (MIMIT), University of Rome Tor Vergata, 00133 Rome, Italy; 5 Department of Systems Medicine, University of Rome Tor Vergata, 00133 Rome, Italy; 6 Center for Musculoskeletal Surgery, Charité-Universitätsmedizin Berlin, Berlin Institute of Health, Corporate Member of Freie Universität Berlin, Humboldt-Universität zu Berlin, 13353 Berlin, Germany

**Keywords:** phage therapy, biofilm, *Staphylococcus aureus*, staphylococcal bacteriophages, antibiotic resistance, prosthetic infections, *Galleria mellonella*, K-wire

## Abstract

*Staphylococcus aureus* implant-associated infections are difficult to treat because of the ability of bacteria to form biofilm on medical devices. Here, the efficacy of Sb-1 to control or prevent *S. aureus* colonization on medical foreign bodies was investigated in a *Galleria mellonella* larval infection model. For colonization control assays, sterile K-wires were implanted into larva prolegs. After 2 days, larvae were infected with methicillin-resistant *S. aureus* ATCC 43300 and incubated at 37 °C for a further 2 days, when treatments with either daptomycin (4 mg/kg), Sb-1 (10^7^ PFUs) or a combination of them (3 x/day) were started. For biofilm prevention assays, larvae were pre-treated with either vancomycin (10 mg/kg) or Sb-1 (10^7^ PFUs) before the *S. aureus* infection. In both experimental settings, K-wires were explanted for colony counting two days after treatment. In comparison to the untreated control, more than a 4 log^10^ CFU and 1 log^10^ CFU reduction was observed on K-wires recovered from larvae treated with the Sb-1/daptomycin combination and with their singular administration, respectively. Moreover, pre-infection treatment with Sb-1 was found to prevent K-wire colonization, similarly to vancomycin. Taken together, the obtained results demonstrated the strong potential of the Sb-1 antibiotic combinatory administration or the Sb-1 pretreatment to control or prevent *S. aureus*-associated implant infections.

## 1. Introduction

Indwelling medical devices, such as cardiac and orthopedic implants, are increasingly used in modern medicine to replace a compromised biological function or missing anatomical structure [1]. However, a major drawback is the risk of developing device-related infections, which represent a devastating complication linked to high morbidity and costs for healthcare systems. Implant-associated infections are caused by sessile-growing microorganisms colonizing the device surface and embedding in an extracellular self-produced polymeric matrix, a complex three-dimensional structure called biofilm [1]. Among different bacteria causing implant-associated infections, *Staphylococcus aureus* is one of the leading pathogens frequently involved in device colonization. *S. aureus* is responsible for 35.5% of prosthetic joint infections (PJIs) [2]. Moreover, most of the *S. aureus* strains isolated in PJIs display biofilm-forming ability [3]. Owing to their structural and biochemical features (e.g., low permeability of the biofilm matrix, altered bacterial physiology and metabolism, presence of persister cells, horizontal gene transfer-mediated antibiotic resistance), *S. aureus* biofilm is refractory to antimicrobial compounds and the host immune system, making the eradication of implant-associated infections rather difficult [4]. Successful antibiotic treatments require the use of bactericidal drugs acting on both persister (low-metabolically active) and biofilm-embedded cells adhering to the device surface. Thus, it remains the main challenge in a clinical setting. For the treatment of PJIs caused by methicillin-sensitive *S. aureus* (MSSA), the intravenous administration of oxacillin or cefazolin is the recommended treatment option [5]. If the infection is due to methicillin-resistant *S. aureus* (MRSA), the treatment includes vancomycin or daptomycin. Moreover, rifampin is added to the above-mentioned antibiotics to treat MRSA infections in patients undergoing debridement with retention or implant re-implantation in one- or two-stage exchange [6]. As the use of rifampin rapidly selects resistant *S. aureus* strains [7], a progressive reduction in its use is desirable. In the last ten years, the use of bacteriophages has re-emerged as a promising antimicrobial therapy for the treatment of biofilm-associated infections due to either Gram-positive or Gram-negative pathogens [8]. Staphylococcal Sb-1 is one of the most active mono-phage formulations against *S. aureus*, including antibiotic-resistant isolates [9]. In our previous studies, we showed that Sb-1 is able to increase the survival of *Galleria mellonella* larvae by preventing or treating MRSA infection compared to the untreated control [10]. Moreover, Sb-1 is also able to target and destroy biofilm-embedded MRSA in vitro by degrading the biofilm matrix and killing persister cells [11]. Notably, Sb-1 displays in vitro synergistic activity with different classes of antibiotics, mostly active against bacterial biofilms. In particular, the best synergistic in vitro activity was observed against MRSA biofilm, sequentially treated first with phages and then with antibiotics [11].

Here, we evaluated the in vivo efficacy of Sb-1 alone and in sequential combination with daptomycin against MRSA ATCC 43300 by using a foreign-body model of *G. mellonella* larvae implanted with short stainless steel Kirschner-wires (K-wires). The phage–antibiotic combination determined a strong reduction in bacteria colonizing the K-wires in comparison to the single treatment administration. In addition, the in vivo ability of Sb-1 to prevent implant-associated infection due to MRSA in comparison to vancomycin was also investigated in the same *G. mellonella* model, showing comparable results between the prophylactic effect of phage and antibiotic.

## 2. Results

### 2.1. Bactericidal Activity of Sb-1 against MRSA ATCC 43300 Colonizing Stainless Steel K-Wires In Vitro

**Figure 1:**
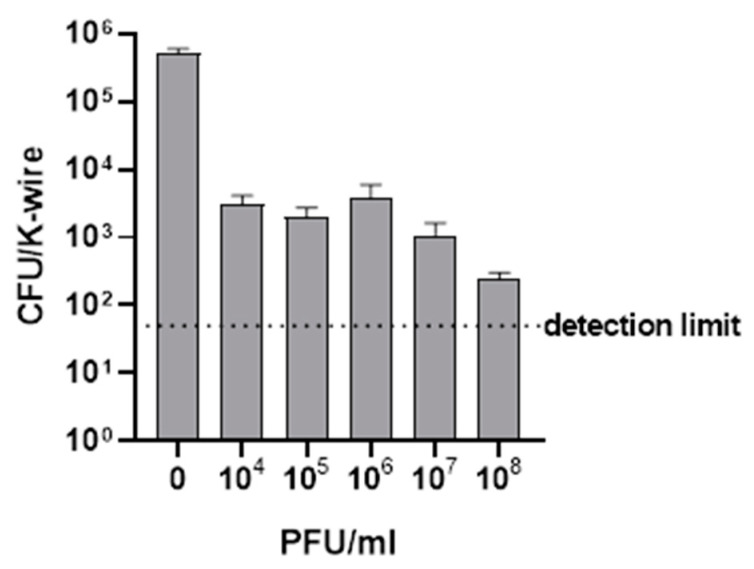
Evaluation of the Sb-1 bactericidal activity against biofilm-embedded MRSA ATCC 43300. The histograms represent the mean CFU number ± SEM of biofilm dislodged MRSA treated/untreated with different titers (PFU/mL) of Sb-1. Dashed line indicates 3 log^10^ CFU reduction in comparison to the untreated control (0). Three independent experiments were performed.

*S. aureus* is prone to colonize and form biofilm on medical implants, including stainless steel devices [12]. To evaluate the potential anti-biofilm activity of Sb-1 phage, a 24 h-old biofilm was formed on the K-wire metal. Then, K-wires were incubated with Sb-1 at different titers (ranging from 10^4^ to 10^8^ PFU/mL) at 37 °C. After 24 h incubation, the number of MRSA ATCC 43300 CFUs that remained on the material was determined. Sb-1 treatment of biofilm samples resulted in a reduction of ≈2 log^10^ in the MRSA ATCC 43300 CFUs compared to the untreated control (no phage), even at the lowest titer tested (Figure 1). However, only the highest titer tested (10^8^ PFU/mL) was able to reduce the CFU number by more than 3 log^10^ compared to the untreated control, defined as the minimal biofilm bactericidal titer according to our previous works [10,13].

### 2.2. Development of G. mellonella Model of Implant-Associated Infection

**Figure 2:**
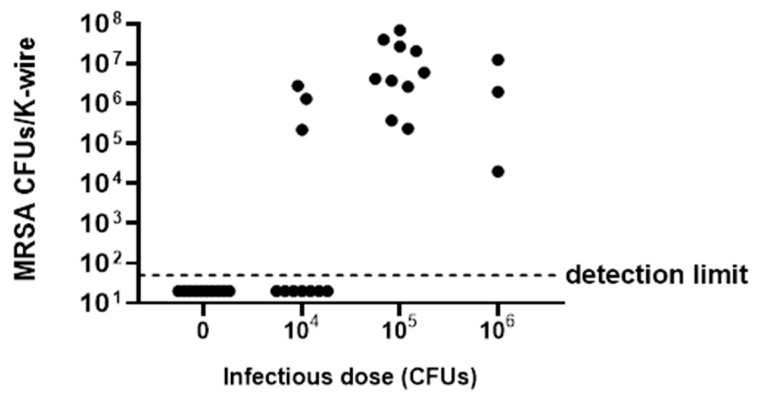
Evaluation of the optimum infectious dose for K-wire colonization in *G. mellonella* infection. Different inocula (0, 10^4^, 10^5^ and 10^6^ CFUs) of methicillin-resistant *S. aureus* ATCC 43300 were tested to find the proper infectious dose. Fifty CFUs were the detection limit. Data from a representative experiment are reported here.

The in vivo implant-associated *S. aureus* infection model was developed in *G. mellonella* larvae by using thin K-wire (cut in short pieces), which resulted in a suitable implant and was easily insertable into larvae. To assess the optimal MRSA ATCC 43300 infectious dose, allowing K-wire colonization two days after infection, K-wire implanted larvae were inoculated with different bacterial inocula, ranging from 10^4^ CFU to 10^6^ CFU. Figure 2 shows the number of CFUs recovered after sonication from each K-wire removed from infected and uninfected larvae. As expected, no bacteria were detected in the uninfected larvae (control group) injected with PBS. When larvae were injected with 10^4^ CFU MRSA ATCC 43300, only three out of ten larvae tested showed K-wire colonization. Similarly, MRSA ATCC 43300 was found only on three out of ten K-wires implanted in larvae infected with 10^6^ CFU bacteria, while the other seven larvae were dead within 24 h of infection. When larvae were injected with 10^5^ CFU MRSA ATCC 43300, all larvae survived, and K-wires were colonized by bacteria with a median of 5.8 × 10^6^ CFU/K-wire. Thus, 10^5^ CFU was selected as the infectious dose for following in vivo experiments of infection treatment and prevention efficacy based on phages.

### 2.3. Kinetic of Sb-1 Stability in G. mellonella Larvae

**Figure 3:**
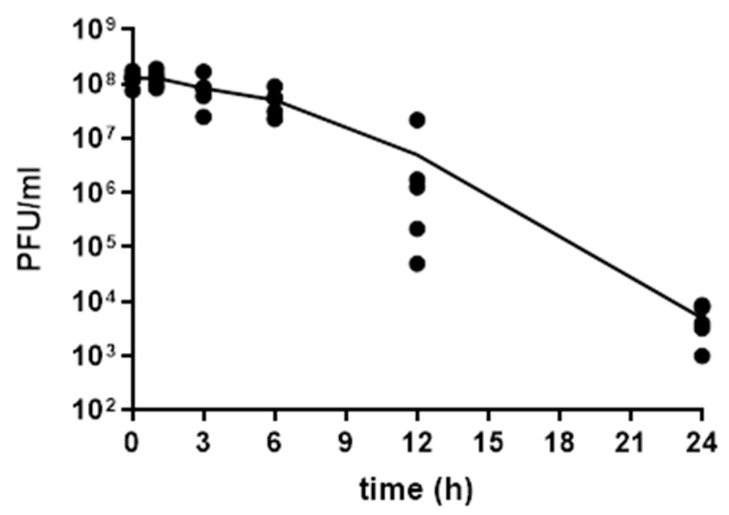
Sb-1 stability in *G. mellonella* larvae over time. Larvae were infected with Sb-1 (10^8^ PFU/mL) and sacrificed at different time points (up to 24 h) to collect hemolymph and perform plaque assays for phage counting. Data from a representative experiment are reported here.

Previous in vivo experiments performed in mice showed that the number of circulating active phages (able to lyse bacteria) reduced over time [14]. A kinetic of Sb-1 titer in larval hemolymph was performed by plaque assay to determine the stability of lytic phages up to 24 h. Once phages (10^8^ PFU/mL) were injected into larvae, five larvae were sacrificed at different time points and hemolymph was collected for phage counting by plaque assay. As shown in Figure 3, a titer between ≈10^8^ PFU/mL Sb-1 was observed for up to 6 h, while after 24 h the titer of phages in larvae was drastically reduced (≈4 log^10^).

### 2.4. Sb-1 Activity versus MRSA ATCC 43300 in G. mellonella Model of Implant-Associated Infection

**Figure 4:**
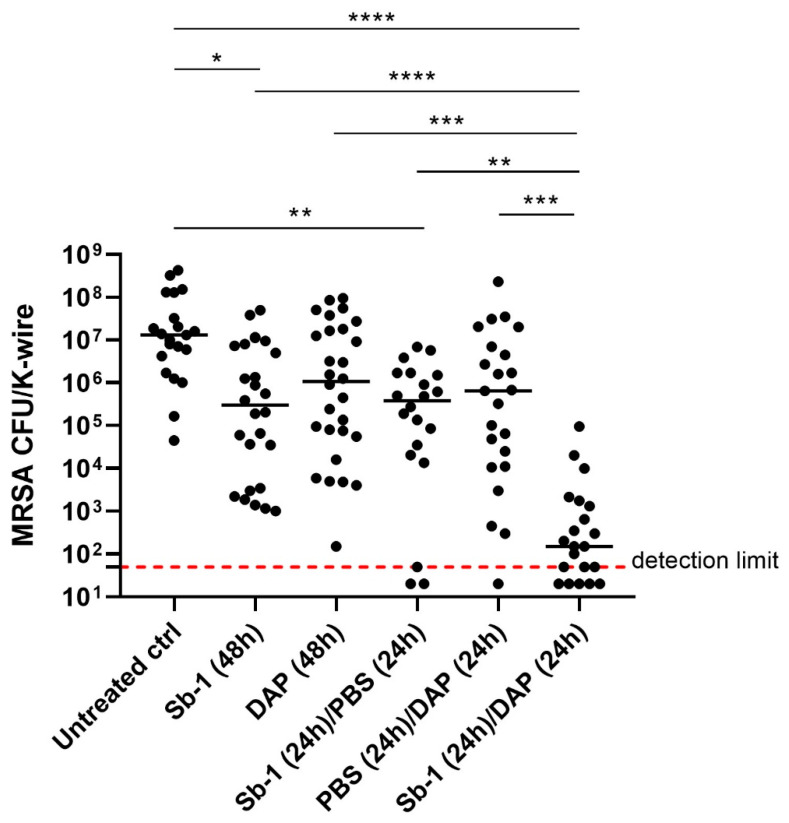
Treatment of MRSA ATCC 43300 K-wire-associated infection with different Sb-1 and daptomycin formulations in *G. mellonella* larvae. After infection with MRSA ATCC 43300 (10^5^ CFUs) larvae were treated with (i) Sb-1 (10^7^ PFUs) alone, (ii) daptomycin (4 mg/kg) alone and staggered administration of (iii) Sb-1 (10^7^ PFUs)/PBS, (iv) PBS/daptomycin (4 mg/kg), and (v) Sb-1 (10^7^ PFUs)/daptomycin (4 mg/kg). Statistical differences in bacterial count per K-wire obtained from different treatment groups were determined by the Kruskal–Wallis test using Dunn correction (* *p* ≤ 0.05; ** *p* ≤ 0.01; *** *p* ≤ 0.001; **** *p* < 0.0001). Data obtained from three independent experiments are reported.

*S. aureus* is known to easily colonize and form biofilm on medical indwelling devices, resulting in an infection that is difficult to treat with current antibacterial therapies. In order to evaluate the ability of Sb-1 (alone or in combination with daptomycin) to reduce the in vivo bacterial colonization of K-wires, larvae were infected with 10^5^ CFU MRSA ATCC 43300 and treated three times/day for 2 days with 10^7^ PFUs Sb-1 and/or 4 mg/kg daptomycin. In these assays, daptomycin was selected for combinatory treatment, as it is the antibiotic currently used in combination with rifampin for the management of MRSA implant-associated infection [6]. Figure 4 shows the CFU number of MRSA ATCC 43300 dislodged by each K-wire removed from larvae treated with either Sb-1 or daptomycin alone (for 48 and 24 h) and with a sequential combination of Sb-1 for 24 h, followed by daptomycin for a further 24 h. The CFU number from K-wires implanted in untreated larvae was also evaluated. A statistically significant reduction of ≈1–1.5 log^10^ CFUs of medians was observed when larvae were treated with Sb-1 alone for either 48 or 24 h. Similar results were obtained when larvae were treated with daptomycin, suggesting that Sb-1 had a lytic activity similar to that exhibited by the antibiotic. A reduction of more than 3 log^10^ CFU/K-wire number was observed when larvae were treated with a staggered combination of phages and antibiotic in comparison to phages or antibiotic administered singularly. Moreover, a stronger reduction in CFU/K-wire number ≈ 4 log^10^ was obtained in comparison to the untreated control.

### 2.5. Ability of Sb-1 to Prevent K-Wire Colonization MRSA ATCC 43300 in G. mellonella Model of Implant-Associated Infection

**Figure 5:**
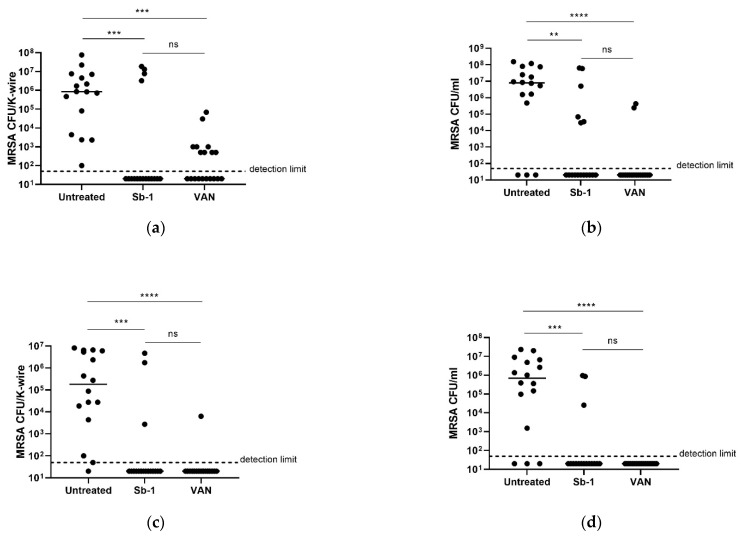
Efficacy of Sb-1 in preventing MRSA ATCC 43300 K-wire colonization in a *G. mellonella* model of implant-associated infection. Sb-1 (10^7^ PFUs) and vancomycin (10 mg/kg) were injected into larvae before the infection with MRSA ATCC 43300 (10^5^ CFUs). The graphs show (**a**) CFU number of K-wires explanted 2 days post-infection from larvae pretreated with either Sb-1 or vancomycin; (**b**) CFU/mL number from hemolymph collected 2 days post-infection from larvae pretreated with either Sb-1 or vancomycin; (**c**) CFU number of K-wires explanted 5 days post-infection from larvae pretreated with either Sb-1 or vancomycin; (**d**) CFU number from hemolymph collected 5 days post-infection from larvae pretreated with either Sb-1 or vancomycin. Statistical differences in bacterial count per K-wire and bacterial count per mL of hemolymph obtained from different pre-treatment groups were determined by the Kruskal–Wallis test using Dunn correction (ns *p* > 0.05; ** *p* ≤ 0.01; *** *p* ≤ 0.001; **** *p* < 0.0001, ns = not significant). Data obtained from two independent experiments are reported.

As an established biofilm is difficult to eradicate, the prevention of the infection is still considered the best strategy to reduce the cases of infection [15]. Therefore, the in vivo ability of 10^7^ Sb-1 in preventing K-wire colonization by MRSA ATCC 43300 was also tested and compared to the efficacy of vancomycin (10 mg/kg), the antibiotic used for the prevention of surgical site infection (10). Prophylactic treatment with either phages or antibiotics was administered as a single dose in implanted larvae 1 h earlier than MRSA ATCC 43300 infection. At two and five days post-infection, larvae were sacrificed and for all of them the K-wire was removed for the MRSA CFU count. In parallel, the hemolymph was collected to evaluate the number of circulating bacteria. Figure 5 shows the CFU number of MRSA ATCC 43300 obtained by recovery from K-wires and in the hemolymph. After 2 days (Figure 5a,b) and 5 days (Figure 5c,d) post-infection, a statistically significant reduction in MRSA ATCC 43300 CFU numbers was observed for either K-wires (Figure 5a,c) or hemolymph (Figure 5b,d) samples recovered from larvae pre-treated with either Sb-1 or vancomycin, in comparison to the untreated controls. In most of the samples, MRSA ATCC 43300 colonies were not detected from K-wires or the hemolymph. Moreover, no statistically significant reduction in MRSA ATCC 43300 CFUs was observed between the two pre-treatment groups.

## 3. Discussion

The increase in antibiotic resistance and the lack in the pipeline of antimicrobial molecules with novel mechanisms of action make bacterial infections increasingly difficult to treat [16]. Moreover, the ability of bacteria, including *S. aureus*, to colonize materials and to form biofilm on implanted medical devices represents a further challenge for the treatment of chronic staphylococcal infections, as only a few antibiotics administered in combination are active against sessile bacteria [17], especially in the case of MRSA strains. Bacteriophage therapy is the most promising antibacterial option to treat chronic infections due to multi-drug-resistant bacteria [8]. In this study, we described the efficacy of the commercially available phage formulation Sb-1 to control/prevent staphylococcal colonization and infection in vivo in a *G. mellonella* model of larvae implanted with stainless steel K-wires. In recent years, *G. mellonella* larvae have been proposed as a suitable in vivo model for investigating the virulence of different microorganisms, including human pathogens, and for evaluating the toxicity and efficacy of antimicrobial compounds [18]. Mostly, larval infection occurred with circulating bacteria and few papers have reported colonization on implanted devices [19,20].

First, we observed the ability of MRSA ATCC 43300 to colonize K-wires in vitro and we confirmed the lytic ability of Sb-1 versus sessile cells *in vitro*. After 24 h incubation, 10^8^ PFU/mL Sb-1 resulted in a bactericidal effect against the staphylococcal biofilm. These results were consistent with data that we observed in previous studies where Sb-1 showed a characteristic antibiofilm activity, strongly reducing CFU/mL numbers of sessile bacteria, degrading the matrix and targeting metabolically active and persister cells of MRSA ATCC 43300 biofilm formed on porous glass beads [10,11].

Due to their use in surgery, together with their small dimension and the ability of MRSA ATCC 43300 to colonize and grow on it, stainless steel K-wires were considered a good implant candidate to be used in *G. mellonella* larvae for the following in vivo experiments. An inoculum of 10^5^ CFUs allowed the in vivo K-wire colonization and larval survival. By contrast, a higher tested inoculum of MRSA (10^6^ CFUs) was shown to be fatal for larvae at 24 h post-infection, as previously reported by the authors [10] and other research groups [21,22]. Thus, we have established a *G. mellonella* model of implant-associated infection caused by MRSA ATCC 43300, which might be used not only to test novel compounds, but also to investigate and provide new insights into the dynamic of the in vivo colonization and biofilm formation.

A critical point for the in vivo treatment of infected K-wire was the phage titer/doses and the number of doses/days to be administered to infected larvae. As we had no previous information regarding phage pharmacokinetics in larvae, the number of phages circulating in the hemolymph of uninfected larvae at different time points after injection was evaluated. The kinetic of clearance and/or inactivation of Sb-1 in larvae showed that the number of lytic phages decreased at 6–12 h post inoculum. Therefore, we decided to administer phages three times/day in order to keep a constant phage titer circulating in the haemolymph. Three times/day was also the dose reported for the phage treatment of patients suffering from prosthetic joint infections due to *P. aeruginosa* biofilm [23]. In agreement with our in vitro results, the lytic activity of Sb-1 versus cells of *S. aureus* ATCC 43300 attached to a material was also observed in *G. mellonella* larvae implanted with K-wires. A group of larvae was treated with daptomycin, which was used as the control of standard treatment, as it is considered a first-choice drug in the clinical practice for methicillin-resistant *S. aureus* biofilm infection [5,6]. A similar CFU reduction was observed in both groups of larvae treated with Sb-1 or daptomycin in comparison to the untreated group, suggesting that phage or antibiotic treatments do not differ in the bacterial killing potential.

Based on our previously published evidence [11] showing that a sequential phage/antibiotic-based treatment of in vitro biofilm determined the eradication of *S. aureus* sessile cells, even at a lower concentration of antibiotic, we verified that the same efficacy also occurred in vivo. The strong reduction in viable *S. aureus* cells observed in this study in the group of larvae sequentially treated with Sb-1 and daptomycin confirmed the bactericidal activity of the Sb-1- daptomycin combinatory treatment previously observed in vitro [11], and it emphasizes the high anti-bacterial efficacy of sequential phage/antibiotic-based treatments. The synergistic effect of phages and antibiotics has been described by different research groups reporting data based on in vivo and in vitro experiments [11,24], but only a few studies investigated the effect of phage/antibiotic staggered administration. The finding that Sb-1 is able to degrade biofilm matrix and target persister cells in vitro [11] suggests a possible explanation for the high in vivo bactericidal efficiency of the sequential Sb-1-daptomycin administration. It can be speculated that the Sb-1-dependent biofilm matrix degradation might enhance the following inhibitory/eradicating effect of the antibiotic drug on bacteria colonizing implants. However, *S. aureus* is known to rapidly develop resistance to rifampin [17]. For this reason, Sb-1 might be used alone for the treatment of sensitive *S. aureus* biofilm, and in combination with daptomycin, as an alternative to the RNA Polymerase inhibitor drug, for the treatment of MRSA infection.

Antimicrobial prophylaxis is currently recommended during orthopedic surgery in which a device is implanted. This consists in the administration of first- or second-generation cephalosporin in all patients, and a supplemental vancomycin administration in MRSA carriers [25].

The results obtained suggested that the fast lysis of methicillin-resistant *S. aureus* by Sb-1 decreases the initial bacterial load, and consequently it reduced the chance of bacteria to colonize the implanted K-wire and establish an infection in *G. mellonella* larvae. Similar results were also obtained for vancomycin, suggesting that Sb-1 might also be used in human therapy as a prophylaxis of surgical site infection instead of antibiotics. In general, phages have a rather narrow host range even at strain level; however, Sb-1 has been shown to be active against ≈90% of diverse global *S. aureus* isolates [9]. Therefore, a formulation of a cocktail containing one or two phages, covering the remaining 10% of resistant strains, might be developed for its prophylactic use to prevent implant-associated infections due to *S. aureus*.

In conclusion, the potential activity of Sb-1 treatment in combination with antibiotics against *S. aureus* cells colonizing stainless steel implants has been demonstrated in vivo. This evidence might spur the design and roll-out of clinical trials to validate the efficacy of phage-based treatments as an adjuvant of antibiotic therapy for the eradication of implant-associated infection due to drug-resistant *S. aureus*.

## 4. Materials and Methods

### 4.1. Bacterial Strain

The methicillin-resistant laboratory strain *S. aureus* ATCC 43300 was used in this study. Bacteria were stored in a cryovial bead preservation system (Roth, Karlsruhe, Germany) at −80 °C and cultivated on Brain-Heart Infusion (BHI; Sigma-Aldrich, Saint Louis, MO, USA) agar plates. Brain-Hearth Infusion broth was used for bacterial inoculum and phage production. Biofilm sonication fluids from larval infection experiments were plated on mannitol salt agar (Sigma-Aldrich, Saint Louis, MO, USA).

### 4.2. Sb-1 Bacteriophage

A commercially available formulation of Staphylococcal bacteriophage Sb-1 (Eliava Biopreparations, Tbilisi, Georgia) was obtained as a 10 mL liquid solution. *S. aureus* ATCC 43300 was used as host to amplify Sb-1. Briefly, an overnight culture of *S. aureus* ATCC 43300 was diluted 1:100 in fresh medium with 0.1 M MgSO_4_ (Sigma-Aldrich, Saint Louis, MO, USA) and incubated at 37 °C until it reached 0.2–0.3 optical density (OD) corresponding to ≈ 1 × 107 CFU/mL for *S. aureus*. Then, bacterial culture was infected with Sb-1 at a multiplicity of infection (MOI) of 1:10. ODs were monitored at different time points until the values reached the OD values obtained before phage infection. Bacterial cultures were centrifuged (4000× *g* for 15 min at 4 °C) and the supernatant was filtered through a 0.22 µm syringe filter (Corning, NY, USA). The phage titer was determined by plaque assay.

### 4.3. Phage Plaque Assay

Phage lysate was diluted in phosphate-buffered saline (PBS; 137 mM NaCl, 2.7 mM KCl, 10 mM Na_2_HPO_4_ and 2 mM KH_2_HPO_4_, pH 7.5) by 10-fold serial dilutions in order to count plaque numbers. Briefly, 0.5 mL bacteriophage lysate of each dilution and 0.3 mL host bacteria from an overnight culture were added to 3 mL top agar (BHI with 0.75% bacteriological agar) at 50 °C and immediately poured onto Brain-Heart Infusion agar plates. Two plates for each dilution were used. After overnight incubation of plates at 37 °C, plaques were counted and the titer was calculated as plaque-forming unit (PFU)/mL. Phages were stored at 4 °C.

### 4.4. In Vitro Biofilm Formation of MRSA ATCC 43300 on Kirschner-Wires and Lytic Activity of Sb-1

Stainless steel Kirschner-wires (K-wires) (DePuy Synthes, Oberdorf, Switzerland) (0.6 mm diameter) were cut into 4 mm length pieces and sterilized by autoclaving. Then, *S. aureus* ATCC 43300 biofilm was allowed to form on their surface at 37 °C in BHI broth supplemented with 0.25% (*w*/*v*) glucose (Sigma-Aldrich, Saint Louis, MO, USA). After 24 h incubation, K-wires were rinsed 3 times using sterile PBS to remove planktonic bacteria. For CFU counting, biofilm-embedded bacteria were dislodged from K-wires by placing each of them in a 2 mL Eppendorf tube with 1 mL PBS and treating them by sequential steps of vortexing (30″), sonicating (1′) and vortexing (30″), as previously reported [26]. The obtained sonication fluid was 10-fold serially diluted and 20 µL of each dilution was seeded on BHI agar plates and incubated at 37 °C for 24 h for colony counting. For the evaluation of phage lytic activity, samples of S. aureus ATCC 43300 biofilms formed on K-wires were incubated at 37 °C for 24 h with the 10-fold serial dilutions of bacteriophages (ranging from 10^8^ PFU/mL to 10^4^ PFU/mL). *S. aureus* biofilm incubated with an equal volume of PBS served as control. After incubation with Sb-1, K-wires were rinsed 3 times with PBS to remove phages and unattached bacteria. For colony counting, samples were sonicated and plated as previously described.

### 4.5. Galleria Mellonella Model of Implant-Associated Infection

**Figure 6:**
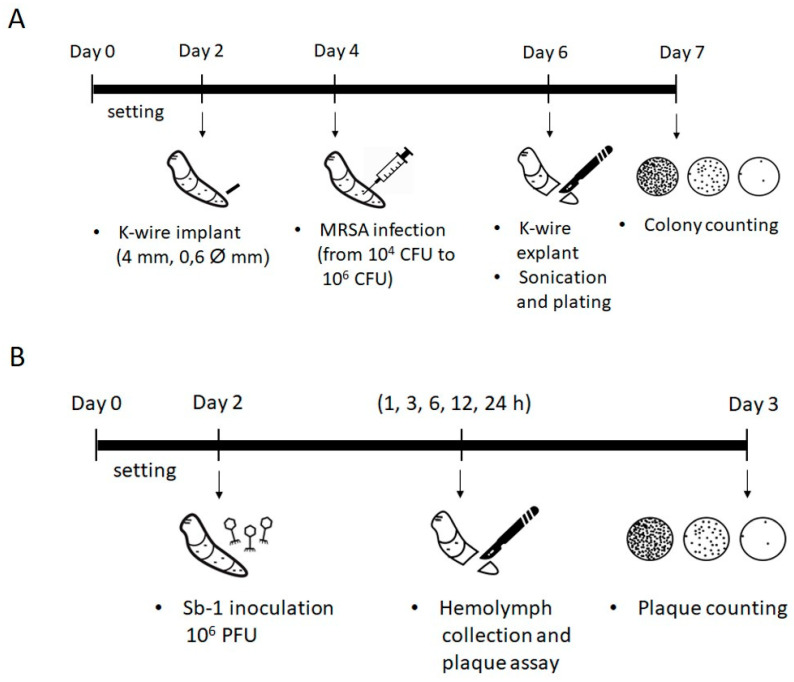
Schematic representation of the experimental model for the establishment of implant-associated infection in *G. mellonella* larvae. (**A**) Evaluation of the optimum infectious dose of MRSA ATCC 43300 able to colonize K-wires implanted in larvae. (**B**) Sb-1 stability in hemolymph of *G. mellonella* larvae and kinetic of phage inactivation.

For in vivo experiments, larvae of wax moth *G. mellonella* bred without antimicrobials or hormones were obtained from BioSystems Technology Ltd. (Exeter, Devon, UK). On their arrival, larvae were stored at room temperature in the dark to prevent them from turning into pupae and used within 3 days. Stainless steel K-wires (4 mm length, 0.6 mm diameter), thin pins used in orthopedic surgery to stabilize bone fragments, were implanted in the last left proleg of larvae by using an insulin syringe needle to create a hole for the entrance of the pins. Then, larvae were left to set for 2 days at RT before infection (Figure 6A). In order to investigate the ability of Sb-1 to either treat or prevent implant colonization in *G. mellonella*, the optimum bacterial infectious dose, able to colonize the implanted K-wire, was determined. Two days post-implant, larvae were infected with different infectious doses (from 10^4^ CFUs to 10^6^ CFUs; 10 µL; 10 larvae per group) in the last right proleg, on the opposite side where a k-wire was inserted. Two days post-infection, larvae were sacrificed to collect the K-wires, which were sonicated as previously described. Then, sonication fluids were plated for colony counting as reported above. To evaluate the stability of circulating Sb-1 in *G. mellonella*, larvae were injected into the last left proleg with 10^8^ PFU/mL phages and, after that, 5 larvae per group were sacrificed at different time points (1, 3, 6, 9, 12 and 24 h) to collect the hemolymph and to count phage titer by plaque assay (Figure 6B).

### 4.6. Sb-1 Efficacy to Reduce K-Wire Colonization in G. mellonella Model of Implant-Associated Infection

**Figure 7:**
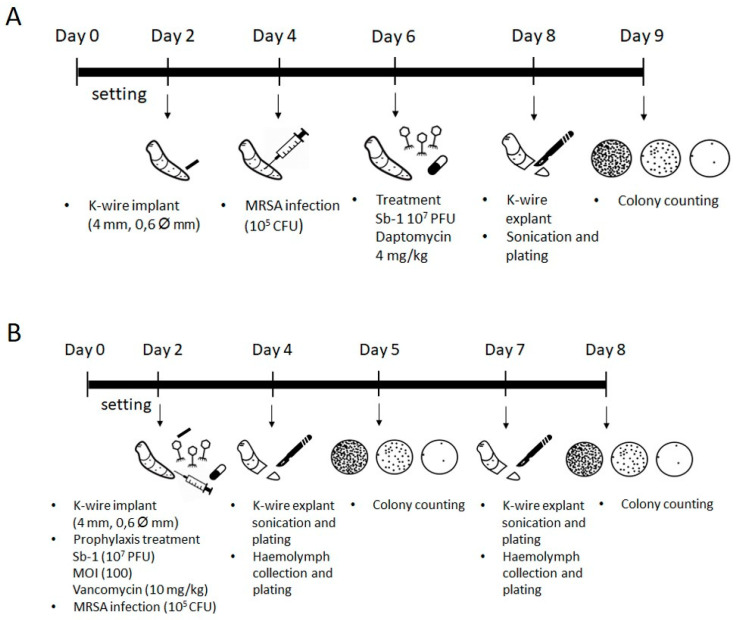
Schematic representation of the procedure used in the experimental model for the evaluation of the Sb-1 in vivo activity in treating (**A**) and preventing (**B**) implanted K-wire colonization by methicillin-resistant *S. aureus* ATCC 43300.

In order to evaluate the ability of phage to treat MRSA biofilm-associated infection, larvae implanted with K-wire and infected with MRSA ATCC 43300, as previously described, were treated with 10^7^ PFUs Sb-1 in 10 µL. Treatments were administered two days post-infection, according to the scheme shown in Figure 7A. Six different groups of treatment (10 larvae each) were included in the study. The treatment was performed three times/day (every 8 h) for 48 h. Larval groups were treated as follows: (i) Sb-1 (10^7^ PFUs) for 48 h, (ii) daptomycin (4 mg/kg) for 48 h, (iii) 10^7^ PFU Sb-1 for 24 h followed by 4 mg/kg daptomycin for further 24 h, (iv) Sb-1 (10^7^ PFUs) for 24 h followed by PBS for further 24 h, and (v) PBS for 24 h followed by Daptomycin (4 mg/kg) for further 24 h. An untreated group of *S. aureus*-infected larvae was included as positive control of biofilm formation. Phage and daptomycin doses were chosen according to their use in the clinical practice [27,28] and previous in vivo experiments [10]. The temporal scheme of treatment was established based on previous results on Sb-1 stability and clinical protocols used for phage treatment of patients with implant-associated infections. Finally, larvae were sacrificed and colony counting of bacteria dislodged from K-wires was performed as reported above. Three independent experiments were performed.

### 4.7. Ability of Sb-1 to Prevent K-Wire Colonization in a G. mellonella Model of Implant-Associated Infection

To evaluate the efficacy of prophylactic application of Sb-1 to prevent MRSA colonization on K-wires, *G. mellonella* larvae (10 for each group) were infected with 10^5^ CFUs MRSA ATCC 43300 on the same day of K-wire implant and 1 h after either Sb-1 (MOI 100) or vancomycin (10 mg/kg) administration according to the procedure showed in Figure 7B. An untreated control group was also included. Then, ten larvae for each group were sacrificed on day 2 and day 5 post-infection, respectively. K-wires were collected and treated as previously described. Hemolymph from all larvae was also collected and serially diluted for plating and colony counting. Two independent experiments were performed.

### 4.8. Statistical Analysis

Statistical analysis of Sb-1 treatment and prevention data obtained from implanted *G. mellonella* larvae was performed by using the non-parametric Kruskal–Wallis test using Dunn’s correction. For all tests, differences were considered significant when *p* values were <0.05. Graphs were plotted and analyzed with Prism software (version 8.0.1; GraphPad Software, La Jolla, CA, USA).

## Data Availability

The original contributions presented in the study are included in the article, further inquiries can be directed to the corresponding author.
